# Meta-Analysis: A Convenient Tool for the Choice of Nose-to-Brain Nanocarriers

**DOI:** 10.3390/bioengineering9110647

**Published:** 2022-11-03

**Authors:** Rania M. Hathout, Eman M. El-Marakby

**Affiliations:** Department of Pharmaceutics and Industrial Pharmacy, Faculty of Pharmacy, Ain Shams University, African Union Organization St., Cairo 11566, Egypt

**Keywords:** nasal, nanocarriers, polymeric, lipid, systematic, meta-analysis

## Abstract

Objectives: The intranasal route represents a high promising route of administration aiming for brain delivery. Yet, it represents one of the most difficult and complicated routes. Accordingly, scientists are in a continuous search for novel drug delivery vehicles such as the lipid and polymeric nanoparticles that are apt to enhance the bioavailability of the administered drugs to reach the brain. In this study, a certain number of publications were selected from different databases and literature. Meta-analysis studies using two different algorithms (DerSimonian–Laird and inverse variance) followed aiming to explore the published studies and confirm by evidence the superiority of nanocarriers in enhancing the brain bioavailability of various drugs. Furthermore, the quantitative comparison of lipid versus polymeric nanosystems was performed. Methods: The area under the curve (AUC) as an important pharmacokinetic parameter extracted from in vivo animal studies was designated as the “effect” in the performed meta-analysis after normalization. Forest plots were generated. Key findings and Conclusions: The meta-analysis confirmed the augmentation of the AUC after the comparison with traditional preparations such as solutions and suspensions. Most importantly, lipid nanoparticles were proven to be significantly superior to the polymeric counterparts.

## 1. Introduction

Several drug delivery researchers and formulators have attempted the nose-to-brain drug delivery as it offers several merits and advantages. The ease of administration, noninvasiveness, proximity to the target organ (the brain), and many other benefits such as the circumvention of one of the hardest barriers for drugs to penetrate, viz. the blood–brain barrier, together with the first-pass effect (liver metabolism) avoidance [1,2] are among the most important pros of this route of administration. The last two aforementioned advantages specifically lead to a remarkable increase in the drug brain bioavailability compared with other conventional pathways of drug administration such as the oral and intravenous routes [3].

To enhance drug absorption and permeability through the nasal mucosa and blood–brain barrier, several attempts have been adopted. Among these attempts are the use of permeation enhancers and drug nanocarriers. The correct selection of the nanoparticulate materials can provide a very successful means of delivering the drug molecule to the brain via the nose. Exploiting the trigeminal and olfactory nerves for the nose-to-brain delivery warrants the usage of several hydrophobic or amphiphilic nanosystems such as the solid lipid nanoparticles, nanostructured lipid carriers, lipid nanocapsules, liposomes, microemulsions, PLGA, Pullulan and chitosan polymeric nanoparticles, and gelatin as protein nanocarriers [4,5,6].

Furthermore, the controlled manner of delivery of drugs currently poses a subject of high significance at both the industrial and academic levels due to its great benefits in the therapy of serious diseases [7,8,9,10]. In a previous and recent meta-analysis study, lipid-based nanosystems were proven to be more capable of increasing the bioavailability of oral drugs after the comparison with conventional systems and formulations [11]. More recently, the use of polymeric nanoparticles was also proven to significantly increase the bioavailability of the aforementioned drugs despite its different nature and chemical structure [12]. Regarding the nose-to-brain delivery, both carrier types have their pros and cons. The lipid vehicles’ strongest advantage lies in the high affinity to the neurons and blood–brain barrier (BBB). On the other hand, the polymeric counterparts are more stable, robust, and easily modulated and conjugated [6,13,14,15].

Systematic reviewing is concerned with the obtainment of empirical evidence from some predetermined eligibility criteria aiming to answer a certain research inquiry. Informatics scientists consider systematic reviewing as a qualitative kind of informatics tool. Nevertheless, meta-analysis is considered its associated quantitative informatics synthesis tool [16]. Meta-analysis is a highly advanced statistical technique that combines the data originating from several studies and extracted from multiple sources. It augments the precision and accuracy of the research studies’ results and outcomes. Moreover, it gives profound postulations and predictions. Meta-analysis is currently accepted as a highly robust informatics tool and a very important method for analyzing and extracting important information from the available literature after data normalization [17]. Moreover, meta-analyses pose important roles in evidence-based healthcare-related issues. Meta-analysis is superior to the other types of studies such as case controls, case reports, cohort studies, and randomized controlled trials. Furthermore, meta-analysis is recognized to be at the top of the pyramid of the levels of evidence [18,19]. Meta-analysis studies offer several advantages. It enhances the statistical power due to sample pooling. Furthermore, this kind of analysis increases the weight of the obtained conclusions. Moreover, meta-analysis is a feasible and economic kind of analysis as it works by the efficient utility of the available treasure of the online resources of databases and literature [20,21,22]. Searching for the correct data and accurately following the eligible criteria are the only hurdles for this method.

Currently, the meta-analysis technique is being integrated in the drug delivery field. Its main benefits lie in comparing novel formulations or advanced drug delivery systems with a conventional counterpart on one hand and comparing several carriers and new delivery systems together on the other hand. It then introduces a new tool for the pharmaceutical industry choice of materials and carriers and helps in decision-making [11,17,23,24].

To this end, systematic reviewing and meta-analysis were used in the current study as pharmaceutics informatics tools [15,25] in order to track the influence of delivering drugs utilizing nanoparticulate systems on the area under the curve (AUC) as an in vivo pharmacokinetic parameter and important indicator of the superior bioavailability of these advanced carriers to the conventional formulations. Furthermore, an additional covariate factor was assessed, specifically the type of the used nanoparticulate system, e.g., lipids such as liposomes, solid lipid nanoparticles, and lipid nanocapsules versus the polymeric such as chitosan, PLGA, Pullulan, and zein.

## 2. Methods

### 2.1. Data Mining

The computerized collection of data was adopted utilizing databases such as Medline^®^ and Embase^®^ and online search engines such as Google Scholar^®^, SCOPUS^®^, ScienceDirect^®^, and Web of Science^®^.

**Figure 1:**
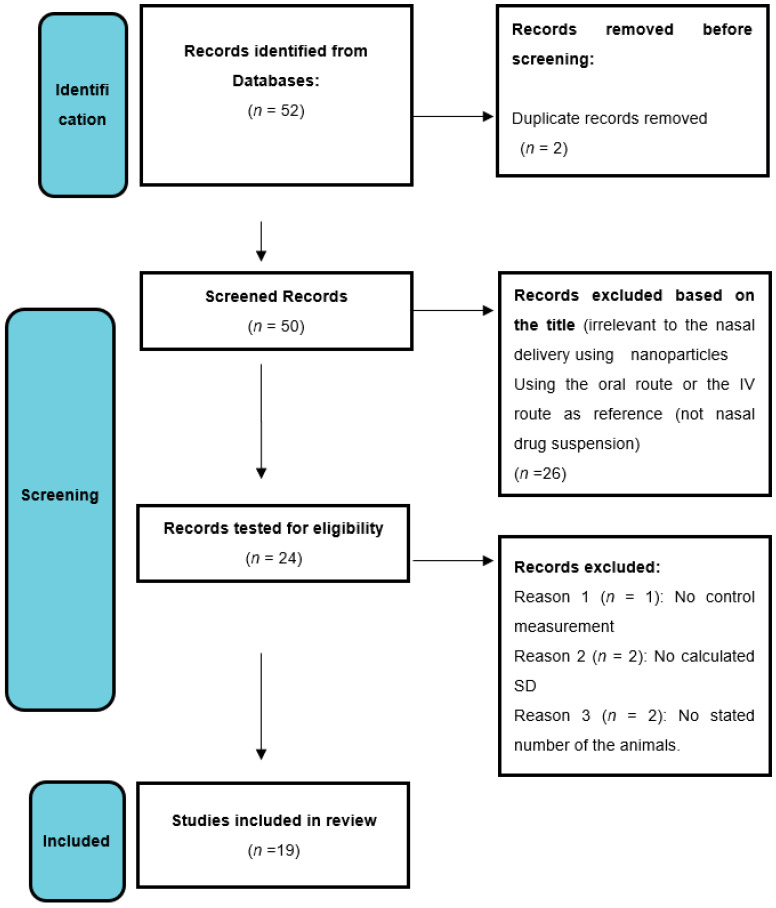
Conducted data-mining process as described in PRISMA guidelines.

The English keywords that were used in the search were as follows: nasal, lipid, polymer, nanocarriers, drug, natural, and synthetic. Processing the literature data and information was performed as described in the guidelines of PRISMA (Preferred Reporting Items for Systematic Reviews and Meta-Analyses: http://www.prisma-statement.org/, accessed on 30 July 2022). The process is depicted in a flowchart diagram illustrated in Figure 1.

### 2.2. Inclusion Data and Criteria

The conducted meta-analysis depended on recording the area under the curve (AUC) as an essential pharmacokinetic parameter. In order for the articles to be accounted as eligible for analysis, these articles should possess certain criteria such as lying in the last ten years (decade), originating from different locations, containing diverse types of lipids and polymers, having a detailed methodology, demonstrating original kind of data, and containing a detailed discussion about the loading of drugs in nanoparticulate systems that are utilized for nose-to-brain delivery. The complete collected eligible articles were additionally extensively screened after the evaluation of the whole and the full text. All of the investigated articles should present original data and should have been published in literature databases as research articles. The mean and standard deviation of the investigated pharmacokinetic parameter, the area under the curve (AUC), should be recorded in the articles. The results of the control group dealing with the investigated drug in all the included studies should be stated. This group should be formulations containing the drug delivered in a conventional formulation via the nasal route. The data that were gathered from the articles adhering to the inclusion criteria were as follows: the name of the studied drug; the author name and year when the study was published; the number of animals utilized for the nanoparticulate system group and, additionally, the conventional formulation group; the type of animal used; the type of the used nanoparticulate system (lipid versus polymeric); and the origin of the material from which the nanoparticulate system is fabricated (natural versus synthetic). The AUC was taken as the bioavailability indicator to compare the drug-loaded nanoparticulate systems and the control (the drug conventional formulation). Various parameters of the adopted meta-analysis are depicted in Table 1.

### 2.3. Meta Analysis

An initial meta-analysis was performed aiming to confirm the bioavailability enhancement effect after loading the intranasal drugs on nanocarriers as depicted by the pharmacokinetic parameter, the area under the curve (AUC), demonstrating the “effect” of the study. Meta-analysis combines the results that are usually obtained from several sources and projects them into a comprehensive conclusive manner. Consequently, the “heterogeneity” was also computed.

OpenMetaAnalyst software (http://www.cebm.brown.edu/openMeta/, accessed on 30 July 2022) was used to feed the two crucial parameters: the effect size, which is the AUC, and the study sample size, which is the number of animals used in the study. The results were meta-analyzed, and the distinguishing charts of this kind of statistical analyses, the forest plots, were provided.

The crucial and solely permissible assumption of the fixed-effect model of meta-analysis stating that the only source of variation between the studies should come from sampling errors (only) was not met in the collected investigated studies of the current meta-analysis due to the variability in the number of used animals in each study. Accordingly, the overall effect size was calculated using the alternative “continuous random-effects model” and utilizing the “DerSimonian–Laird method”.

The results were further confirmed using another software: Review Manager v.5.4.1 (Cochrane Collaboration, London, UK), which utilizes another algorithm, the inverse variance for the overall effect size calculation. The continuous random-effects model was also used in this analysis.

The random-effects model is concerned with the different sources of variability occurring between all the studies. Examples of these sources in the current study include the study year, authors, differently used drugs and their variable doses, conditions of conducting the different studies, type of the utilized animals, origin of the used material, measurements method, and sample size. Therefore, and logically, the random-effects model was claimed suitable for the conducted meta-analysis. Nevertheless, heterogeneity was evaluated using two important statistical parameters: the Q statistic and I^2^ index. The Q statistic provides an account on the presence or, on the contrary, the absence of heterogeneity among a studies’ set that is related to all of the stated variables, whereas the I^2^ index is considered an indicator of the heterogeneity degree. The standardized mean difference (SMD) and its corresponding 95% confidence interval (CI) were computed and presented in the forest plot. The *p*-value was used as an indicator of the presence of significance. The sensitivity and robustness of the study were assessed using the statistical technique leave-one-out meta-analysis.

The effect size was computed through the following equation [12]:

(1) $$E=\frac{I_{AUC}}{N}$$

where *E* represents the effect size, *I_AUC_* is the targeted area under the curve, and *N* is the number of animals used in the investigated study (the sample size).

The standard mean difference (SMD) was also computed as follows:

(2) $$SMD=\frac{Mean_{a}-Mean_{b}}{S_{pooled}}$$

where *S_pooled_* is

(3) $$\sqrt{\frac{\left(n_{a}-1\right)S_{a}^{2\;}+\left(n_{b}-1\right)S_{b}^{2}}{n_{a}+n_{b}-2}}$$

where *n_a_* is the number of animals that received the nanocarrier, *n_b_* is the number of animals that were administered with the control (the drug conventional formulation), *S_a_* is the standard deviation of the nanocarrier mean effect, whereas *S_b_* is the standard deviation of the mean effect of the drug conventional formulation.

The weight of each study was calculated as follows:

(4) $$SW=\frac{1}{SE^{2}}$$

where *SW* is the weight of the investigated study, and *SE* is the standard error of the study.

For optimizing purposes, the studies that possessed odd (whether highest or lowest) weights were removed (ignored), and the results were re-meta-analyzed.

*Q* usually represents the amount of observed heterogeneity, whereas the *I*^2^ index presents the amount of predicted heterogeneity due to chance and is usually considered the quantitative degree of heterogeneity and is computed as follows: $I^{2}=100\times \frac{Q-df}{Q}$, where *df* is the degrees of freedom calculated as the studies number −1.

Moreover, the investigated studies were analyzed according to two subgroup categories. The first category was (a) the polymeric nanoparticulate systems versus (b) the lipid nanoparticulate systems, and the second category included (c) the naturally originated material of the nanoparticulate systems versus (d) the synthetically originated material of the nanoparticulate systems.

It is also worth to note that the publication bias was assessed by constructing a funnel plot using Reviewer Manager v.5.4.1. software.

## 3. Results and Discussion

**Figure 2:**
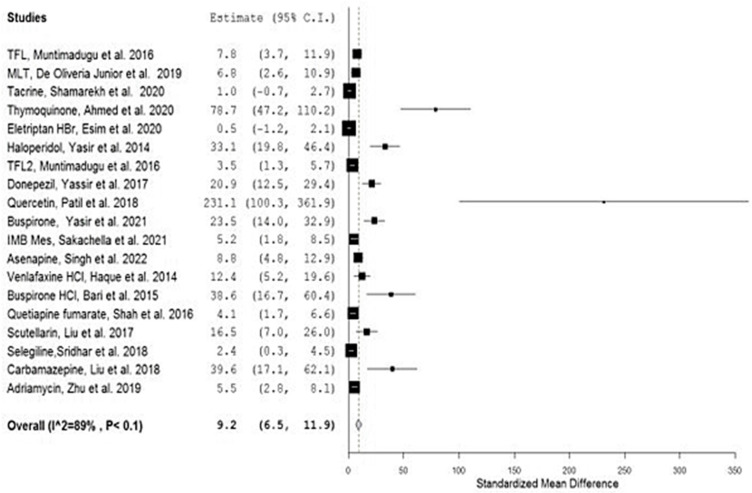
Forest plot presenting meta-analysis of investigated studies [26,27,28,29,30,31,32,33,34,35,36,37,38,39,40,41,42,43,44] generated using OpenMetaAnalyst^®^ software.

**Figure 3:**
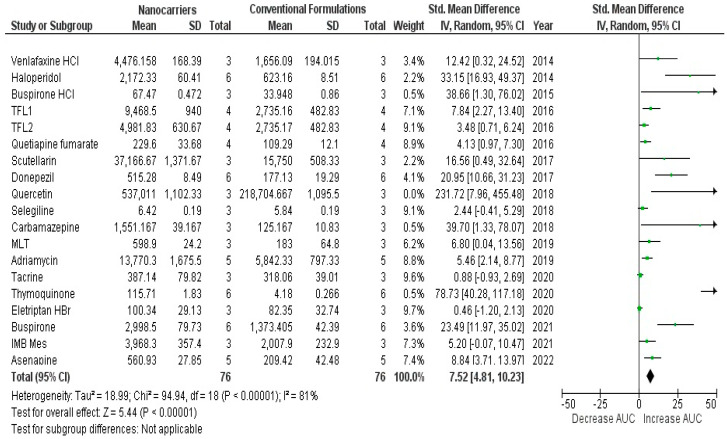
Forest plot presenting meta-analysis of investigated studies [26,27,28,29,30,31,32,33,34,35,36,37,38,39,40,41,42,43,44] generated using Review Manager v.5.4.1.^®^ software.

Table 1 depicts the results that were obtained from the performed meta-analysis after computing the standardized mean difference (SMD) for each of the investigated studies together with its corresponding lower and upper confidence intervals (C.I.s). The presence of the calculated C.I.s on one side of the number zero as a cutoff (i.e., either both confidence intervals are positive or both negative), as illustrated by the software-generated forest plots (Figure 2 and Figure 3), represented by the symbol of a diamond indicating the overall mean not touching the line of no effect (the zero line), implies the significance of all the results of the investigated studies [23,45].

The overall SMD estimate was considered significant as its *p*-values were calculated as <0.1 and <0.00001 and possessed pooled estimates of 9.2 and C.I. (6.5, 11.9) and 7.52 and C.I. (4.81, 10.23) for the results generated using OpenMetaAnalyst and Review Manager, respectively [46]. Since the values of both the upper and lower confidence intervals are higher than zero, the significance of the results was confirmed [47] and proved the existence of a true effect of the used nanocarriers on the nose-to-brain bioavailability of the included drugs as indicated by the area under the curve (AUC) as a very important pharmacokinetic parameter.

The results were validated using the leave-one-out meta-analysis (by ignoring one study at a time and reconducting the analysis). This technique has proven the high sensitivity and accuracy of the outcomes as the pooled estimate ranged from 8.3 to 10.3 for all of the performed and conducted analyses [48].

The mechanism of transport of nanoparticles via the nose-to-brain route happens through the trigeminal or olfactory neurons using endocytotic or neuronal pathways. Laser confocal scanning microscopy has previously proven that the nanocarriers within the range of 20–200 nm can follow the clathrin-coated pits. On the other hand, larger nanoparticles in the size range of 200–1000 nm can be uptaken through the caveolae-mediated endocytosis. The nanoparticles can also be transported from the endothelial cells to olfactory neurons through endocytosis or pinocytosis, and its movement takes place along the neuronal axon. This transport pathway takes place when the nanoparticle size is within the axon diameter, which is 100–700 nm. Accordingly, the intranasal delivery of nanoparticles is considered a promising platform for targeting life-threatening diseases such as the different grades of gliomas. Furthermore, regarding the nanocarrier systems, the most promising nanoparticle classes that are at the focus of research endeavors for brain-targeting are the polymer-based nanoparticles and lipid-based nanoparticulate systems [49].

The heterogeneity score of the meta-analysis was relatively high with an amount (Q) equal to 158.2 and the quantitative degree of heterogeneity (I^2^) scores of 89% and 81% according to the two adopted software packages. There are many sources of heterogeneity that contributed to this value, such as the difference in the year of study, the used animals, the number of these used animals, the used drug, the drug dosage, measurement types, climates and breeding conditions of the animals, and the different laboratories and researchers [50].

The variability in the kind and number of the used animals together with the type of drugs and their dosages specifically has the most significant effect on the weight of each study. Hence, optimizing the study regarding heterogeneity was attempted. The studies with odd weights, viz. possessing weights less than 2%, were excluded [51]: Ahmed et al. 2020, Patil et al. 2018, Bari et al. 2015, and Liu et al. 2018 (Table 2).

**Figure 4:**
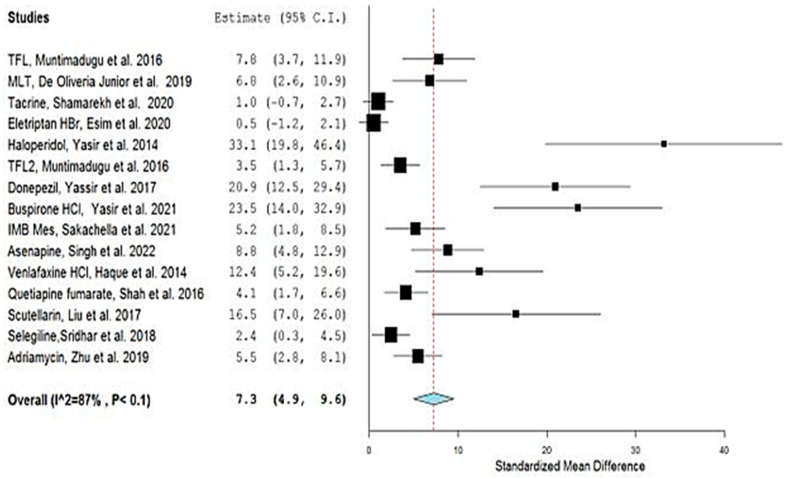
Forest plot of optimized meta-analysis for the studies: [1,2,3,5,6,7,8,11,12,13,14,15,16,17,19], according to OpenMetaAnalyst software.

**Figure 5:**
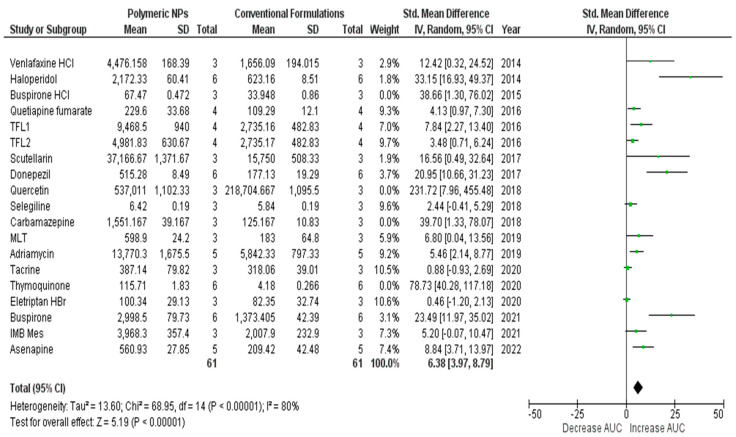
Forest plot of optimized meta-analysis for the studies: [1,2,3,5,6,7,8,11,12,13,14,15,16,17,19], according to Review Manager software.

Consequently, the overall pooled estimate was amended to 7.3 (4.9, 9.6) and 6.3 (3.97, 8.79), and the heterogeneity significantly dropped to an amount of 105.0 and a degree of 87% and 80%, for OpenMetaAnalyst and Review Manager, respectively. This improvement was obvious in more homogenous bullets and confident interval lines in the generated forest plot representing the optimized meta-analysis (Figure 4 and Figure 5).

**Figure 6:**
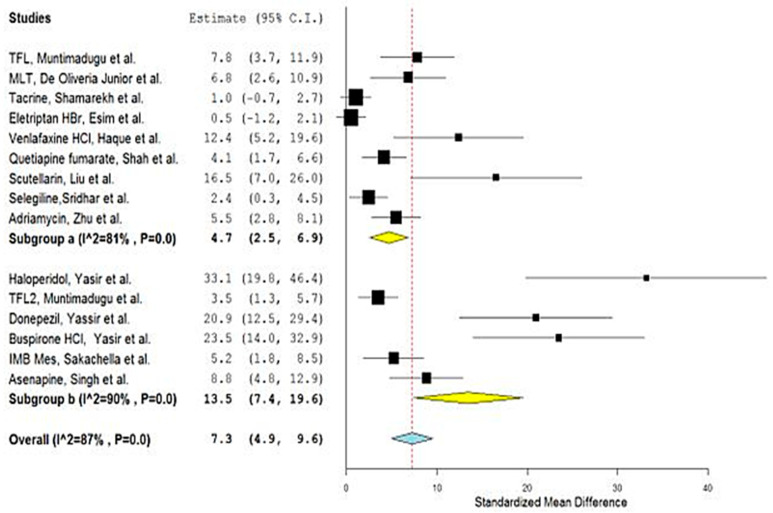
Forest plot of investigated subgroups: (a) polymeric nanoparticulate systems versus (b) lipid nanoparticulate systems for the studies: [1,2,3,5,6,7,8,11,12,13,14,15,16,17,19], (OpenMetaAnalyst results).

**Figure 7:**
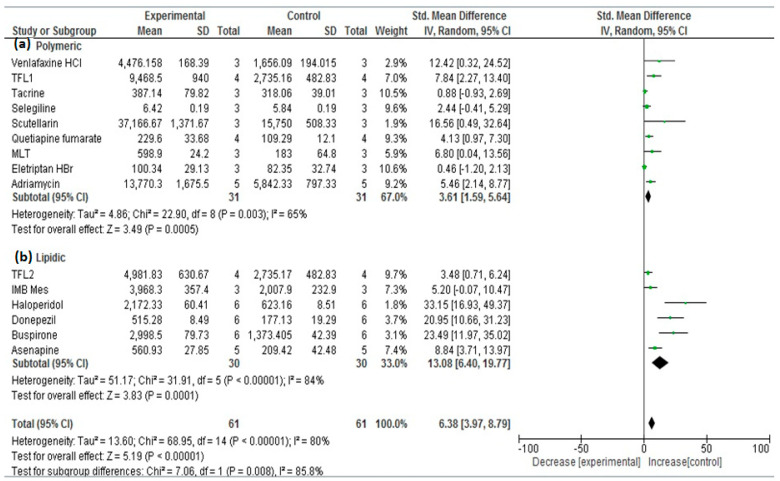
Forest plot of investigated subgroups: (a) polymeric nanoparticulate systems versus (b) lipid nanoparticulate systems for the studies: [1,2,3,5,6,7,8,11,12,13,14,15,16,17,19], (Review Manager results).

Digging more in the literature, the included studies were categorized into two novel subgroups with respect to the type of the material that was utilized to synthesize the nanocarrier or nanoparticulate system—subgroup 1: the polymeric nanoparticulate system encoded as (a); and subgroup 2: the lipid nanoparticulate system encoded as (b). A subgroup meta-analysis was performed where the subgroup (a) pooled estimate score was 4.7 and 3.61 with C.I.s (2.5, 6.9) and (1.59, 5.64) using the two adopted software packages OpenMetaAnalyst and Review Manager, respectively; on the other hand, the subgroup (b) pooled estimate score was 13.5 and 13.08 with C.I.s (7.4, 19.6) and (6.40, 19.77) utilizing the same software packages, respectively. The non-overlapping confidence intervals, which are also illustrated by the borders of the yellow or black diamond symbols generated by the two software packages depicting the two analyzed subgroups in Figure 6 and Figure 7, imply a significant difference between the two subgroups [52]. The superiority of lipid-based nanoparticulate systems in enhancing and increasing the bioavailability of their enclosed drugs compared with the polymeric-based counterparts can be attributed to the higher lipophilic properties of these carriers that exceed those of the polymeric competitors that lead to higher penetration ability into the nasal mucosa and blood–brain barrier, which, in turn, causes better bioavailability. The higher lipophilicity also imparts more affinity for the trigeminal and olfactory nerves, which are key players in the nose-to-brain delivery [53]. This important finding would consequently boost the researchers to concentrate on the type of the material used for the nose-to-brain delivery of drugs and, hence, concentrate on the use of the lipid nanoparticulate systems. Improving the stability of these systems toward oxidation and rancidity would add great assets to their high biocompatibility, safety, and toxicological profiles.

**Figure 8:**
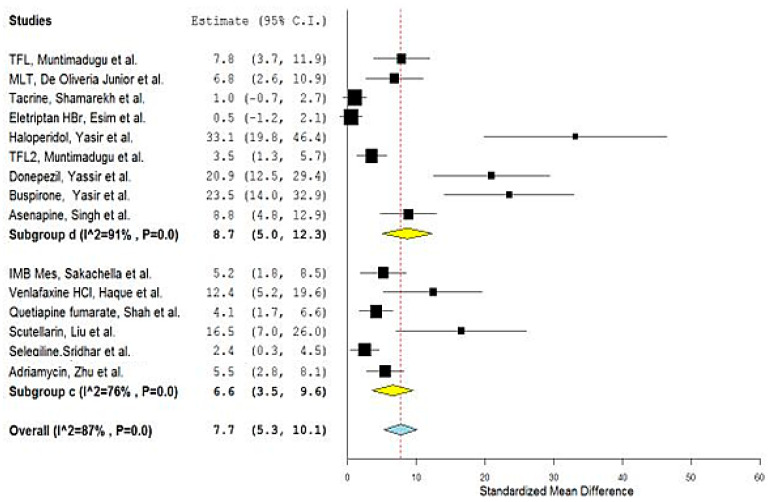
Forest plot of investigated subgroups: (c) naturally originated material of nanoparticulate systems versus (d) synthetically originated material of nanoparticulate system for the studies: [1,2,3,5,6,7,8,11,12,13,14,15,16,17,19], (OpenMetaAnalyst).

**Figure 9:**
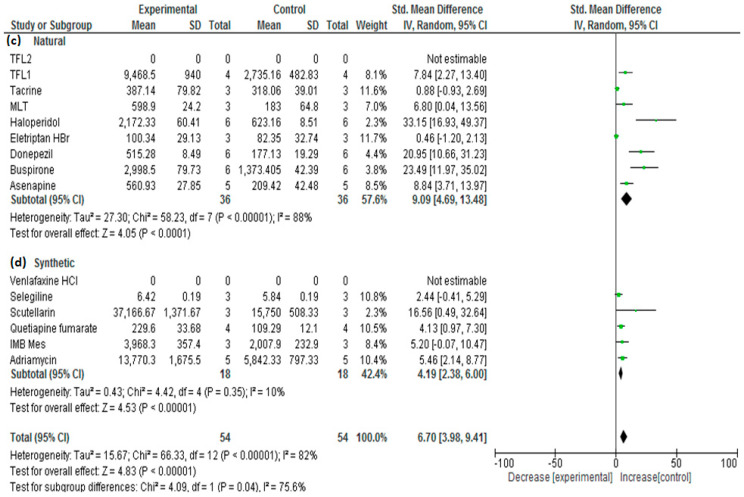
Forest plot of investigated subgroups: (c) naturally originated material of nanoparticulate systems versus (d) synthetically originated material of nanoparticulate system for the studies: [1,2,3,5,6,7,8,11,12,13,14,15,16,17,19], (Review Manager).

Interestingly, comparing the naturally originated nanoparticulate carriers with their synthetically driven counterparts did not show significant differences as demonstrated by the overlapping confidence intervals of the obtained overall means of both subgroups (Figure 8 and Figure 9).

These results, again, encourage the formulators to concentrate on the type and nature of the nanoparticulate carrier material rather than its origin [54].

**Figure 10:**
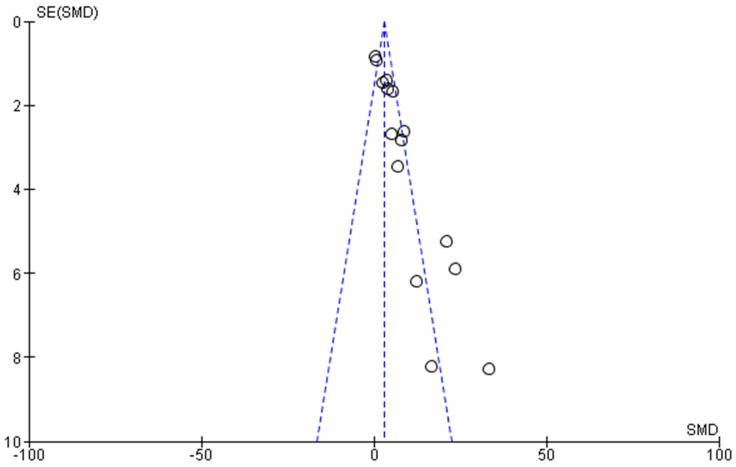
Funnel plot assessing publication bias of investigated studies.

Finally, Figure 10 represents the funnel plot generated from Review Manager v. 5.4.1. software in order to judge the presence of any publication bias. The obtained plot presented a skewed funnel, though most of the studies lie within the acceptable confidence intervals. This may be explained by the fact that, usually, researchers publish their positive results rather than their negative counterparts. In the current case, the researchers are biased to publish articles confirming the augmenting effect of the use of their prepared or synthesized nanoparticles in enhancing the brain bioavailability of drugs that are intranasally administered. Furthermore, the studies that have smaller sizes usually possess higher standard errors (as revealed by the three circular points lying outside the confidence intervals) [55].

It is worth mentioning that, experimentally, the permeation of insulin through the nasal mucosa was recently proven to be superior from solid lipid nanoparticles and outperforming the insulin encapsulated in PLGA nanoparticles by Akel et al., 2021 [56].

It is worth to note that the small data set conforming to the eligibility criteria is one of the limitations of this study. Despite the pooling and normalization effects of the meta-analysis study conduction, increasing the number of the analyzed studies would decrease the heterogeneity and publication bias [57,58].

## 4. Conclusions

The conducted meta-analysis study as a quantitative synthetic statistical tool study confirmed the superiority of the nanoparticulate systems over the conventional formulations systems regarding the drug bioavailability via the nose-to-brain delivery administration route. Moreover, the meta-analysis conducted in this study could prove a useful tool in identifying the optimum type of carriers for this kind of delivery as revealed by the overall standardized mean differences and their associated confidence intervals and as depicted in the forest plots generated by two different algorithm meta-analysis software packages. The optimum nanocarriers were the lipid-based nanoparticulate carrier systems, which outperformed their polymeric counterparts. As a future perspective, this study outcome would encourage scientists and drug delivery researchers to exert more efforts on improving the stability of these important carriers, which represent the main obstacle to their wide use in the medicine market despite their high bio-affinity, biocompatibility, and safety profiles. The stability problem remains the main challenge and hurdle to the development of the advanced and successful nose-to-brain drug delivery systems. No significant differences were obtained between naturally originated and synthetically driven nanoparticulate carriers. This also warrants scientists and formulators to concentrate on the choice of the type and nature of the carriers’ materials rather than their respective origins.

## Figures and Tables

**Table 1:** Meta-Analysis of area under the curve (AUC) in studies of intranasal loaded drugs in lipid and polymeric nanoparticulate systems as compared with their controls (conventional formulations).

No.	Drug	Year	No. of Animals ^1^	Types	Group A ^2^		Nano Carrier Type	Group B ^3^		SMD	Lower/Upper Confidence	Ref.
					NPs Mean AUC ^4^	AUC SD		NPs Mean AUC ^2^	AUC SD			
1	TFL1	2016	4	Sprague–Dawley Rats	9468.50	940.00	PLGA ^a,d^	2735.16	482.83	7.827	3.749/11.905	[26]
2	MLT	2019	3	Wistar rats	598.90	24.20	PCL ^a,d^	183.0	64.80	6.784	2.626/10.943	[27]
3	Tacrine	2020	3	Wister albino rats	397.14	79.82	PLGA ^a,d^	318.06	39.01	1.004	−0.694/2.703	[28]
4	Thymoquinone	2020	6	Rats	115.71	1.83	PLGA ^a,d^	4.18	0.266	78.704	47.196/110.212	[29]
5	Eletriptan HBr	2020	3	Wistar albino rats	100.34	29.13	PLGA ^a,d^	82.35	32.74	0.463	−1.158/2.085	[30]
6	Haloperidol	2014	6	Wistar albino rats	2172.33	60.41	SLN ^b,d^	623.16	8.51	33.138	19.832/46.443	[31]
7	TFL2	2016	4	Sprague–Dawley Rats	4981.83	630.67	SLN ^b,d^	2735.17	482.83	3.475	1.279/5.670	[26]
8	Donepezil	2017	6	Wistar Albino rats	515.28	8.49	SLN ^b,d^	177.13	19.29	20.937	12.485/29.390	[32]
9	Quercetin	2018	3	Wistar rats	537,011	1102.33	NLC ^b,d^	218,704.667	1095.5	231.110	100.340/361.881	[33]
10	Buspirone	2021	6	Albino Wistar rats	2998.50	79.73	SLN ^b,d^	1373.405	42.39	23.485	14.022/32.949	[34]
11	IMB Mes	2021	3	Sprague–Dawley (SD) rats	3968.3	357.4	Liposomes ^b,c^	2007.9	232.9	5.186	1.844/8.527	[35,36]
12	Asenapine	2022	5	Charles Foster (CF) rats	560.93	27.85	NLC ^b,d^	209.42	42.48	8.834	4.769/12.900	[37]
13	Venlafaxine HCl	2014	3	Rats	4476.158	168.39	Alginate ^a,c^	1656.09	194.015	12.387	5.198/19.575	[38]
14	Buspirone HCl	2015	3	Albino Wistar rats	67.47	0.472	Thiolated chitosan ^a,c^	33.948	0.86	38.558	16.683/60.432	[39]
15	Quetiapine fumarate	2016	4	Sprague–Dawley rats	229.6	33.68	Chitosan ^a,c^	109.29	12.1	4.130	1.677/6.582	[40]
16	Scutellarin	2017	3	Mice	37,166.67	1371.67	HP-ß-CD/chitosan ^a,c^	15,750	508.33	16.520	7.037/26.003	[41]
17	Selegiline	2018	3	Rats	6.42	0.19	Chitosan ^a,c^	5.84	0.19	2.436	0.324/4.548	[42]
18	Carbamazepine	2018	3	Mice	1551.167	39.167	Carboxymethyl chitosan ^a,c^	125.167	10.83	39.596	17.136/62.057	[43]
19	Adriamycin	2019	5	Wistar rats	13,770.3	1675.5	Cholesterol-modified Pullulan ^a,c^	5842.33	797.33	5.454	2.762/8.147	[44]

^1^ Both groups had the same number of animals. ^2^ Nose-to-brain formulation; ^3^ Conventional formulation; ^4^ ng.h/mL. Used polymer types were denoted as subgroup “^a^” for polymeric and subgroup “^b^” for lipid. Origin of used polymers was denoted as subgroup “^c^” for naturally originated material and subgroup “^d^” for synthetically originated material. Tfl stands for Tarenflurbil. MLT stands for Melatonin. IMB Mes stands for Imatinib Mesylate.

**Table 2:** Calculated weights of investigated studies.

Study Name	Weights
TFL, Muntimadugu et al.	7.0%
MLT, De Oliveria Junior et al.	7.0%
Tacrine, Shamarekh et al.	8.0%
Thymoquinone, Ahmed et al.	0.7%
Eletriptan HBr, Esim et al.	8.1%
Haloperidol, Yasir et al.	2.8%
TFL2, Muntimadugu et al.	7.9%
Donepezil, Yassir et al.	4.6%
Quercetin, Patil et al.	0.0%
Buspirone, Yasir et al.	4.2%
IMB Mes, Sakachella et al.	7.4%
Asenapine, Singh et al.	7.0%
Venlafaxine HCl, Haque et al.	5.3%
Buspirone HCl, Bari et al.	1.3%
Quetiapine fumarate, Shah et al.	7.8%
Scutellarin, Liu et al.	4.1%
Selegiline, Sridhar et al.	7.9%
Carbamazepine, Liu et al.	1.3%
Adriamycin, Zhu et al.	7.7%

## Data Availability

Data sharing not applicable.
